# Tetracycline Effects on *Candida Albicans* Virulence Factors

**DOI:** 10.1155/2008/493508

**Published:** 2008-05-29

**Authors:** Logan McCool, Hanh Mai, Michael Essmann, Bryan Larsen

**Affiliations:** Infectious Disease Research Laboratory, Ryan Hall 209, Des Moines University, 3200 Grand Avenue, Des Moines, Iowa 50312, USA

## Abstract

*Object*. To determine if tetracycline, previously reported to increase the probability of developing symptomatic vaginal yeast infections, has a direct effect on *Candida albicans* growth or induction of virulent phenotypes. *Method*. In vitro, clinical isolates of yeast were cultivated with sublethal concentrations of tetracycline and yeast cell counts, hyphal formation, drug efflux pump activity, biofilm production, and hemolysin production were determined by previously reported methods. *Results*. Tetracycline concentrations above 150 *μ*g/mL inhibited *Candida albicans*, but at submicrogram/mL, a modest growth increase during the early hours of the growth curve was observed. Tetracycline did not inhibit hyphal formation at sublethal concentrations. Hypha formation appeared augmented by exposure to tetracycline in the presence of chemically defined medium and especially in the presence of human serum. Efflux pump *CDR1* was upregulated and a nonsignificant trend toward increased biofilm formation was noted. *Conclusion*. Tetracycline appears to have a small growth enhancing effect and may influence virulence through augmentation of hypha formation, and a modest effect on drug efflux and biofilm formation, although tetracycline did not affect hemolysin. It is not clear if the magnitude of the effect is sufficient to attribute vaginitis following tetracycline treatment to direct action of tetracycline on yeast.

## 1. INTRODUCTION

Epidemiological studies purport that the use of certain antibiotics during pregnancy increases susceptibility to vulvo-vaginal candidiasis [1, 2]. This condition is generally believed to result from the suppression of a bacterial flora in the face of antibacterial drugs that fail to inhibit *Candida*, resulting in its overgrowth and subsequent emergence of symptoms. Although this concept is often repeated in textbooks, surprisingly little direct evidence exists to support the concept that vaginal candidiasis is caused by prior antibiotic treatment. A report by Burns et al. [3] provided correlation between antibiotic use and vaginal colonization and candidiasis. Since the role of antibiotics in eliciting yeast vaginitis is medical dogma, there has been little reason to expend effort on developing actual data on the relationship between antibiotics and candidiasis.

In 1998, Glover and Larsen [4], curious about the magnitude of the impact of antibiotics on yeast vaginitis, unexpectedly found that nonantibiotic-treated pregnant patients actually became symptomatic more frequently than did the patients treated with antibiotics. This prompted a search in the literature for evidence of a direct effect of antibiotic treatment on yeast colonization. The clearest evidence for antibiotic effects on yeast colonization came from the paper by Caruso [1] who implicated tetracycline as increasing yeast vaginal colonization. Tetracycline was also implicated by Oriel and Waterworth [5] who indicated that 14 days of tetracycline or minocycline treatment increased vaginal *Candida* culture positive rate from 13% to 29%.

Although tetracycline was not employed in Glover and Larsen’s analysis [4] because their study focused on pregnant women, they did speculate that tetracycline may have had some direct effect on *Candida* independent of its effect on competing vaginal bacterial flora. Previous studies showed that estradiol and other drugs were able to affect the growth of *Candida* and the expression of certain virulence-related factors [6, 7], implying that a variety of chemical compounds including tetracycline, may exert a direct effect on *Candida* apart from or in addition to its antimicrobial activity. It is against this background that we investigated whether tetracycline may have direct effects on the growth or expression of virulence attributes of *Candida albicans*. Such a discovery could illuminate the potential relationship of antibiotics, particularly tetracycline, and candidiasis that is distinct from the antimicrobial effect on the normal flora.

## 2. MATERIALS AND METHODS

Human isolates of *Candida albicans* were available in our laboratory culture collection. These were originally obtained from gynecologic specimens and identified as *C. albicans* by observing microscopic morphology, germ-tube formation in human serum, and production of brown colonies on BIGGY agar. *Candida* were maintained at −80°C, and when needed, revived on Sabouraud’s Dextrose Agar and held at 4°C until use in a specific experiment. Prior to any experiment, the organisms were removed from the refrigerated plate culture and subcultured in an appropriate liquid medium to create an actively-growing starter culture.

Six human isolates of *C. albicans* were used. Three of these had previously been shown to have growth stimulation in the presence of estrogen (GT 387, GT 142, GT 397) and the other three showed restricted growth in the presence of estrogen (GT 188, GT 132, 986). In addition, a special indicator organism, CaSA1 [8] which has a green fluorescent protein gene inserted in place of one allele of the *CDR1* structural gene, was used to investigate regulation of *CDR1*. This gene encodes an ATP binding cassette molecular pump that confers multidrug resistance on the organism and is upregulated both by azole antifungal drugs as well as estradiol [9].

Growth yield studies were performed by diluting an overnight starter culture prepared in Yeast Nitrogen Broth with 0.5% glucose (YNB), and the culture was diluted 10^4^-fold in fresh YNB with and without additives at final concentrations as described in the individual experiments. Experiments employed tetracycline or estrogen (1, 3, 5[10]-estratriene-3, 17*β*-diol, referred to hereafter as 17*β*-estradiol) as additives in the growth experiments. 17*β*-estradiol was employed as a positive control in some experiments since we knew from prior studies what effect this compound had on the test strains used. When experimental cultures were prepared, they were incubated overnight at 30°C or 37°C before counts were made by hemacytometer. For consistency, hyphal forms were counted on the basis of the presumed number of nuclei visualized. Budding cells were counted as 2 when a constriction at the bud neck had formed between the mother and daughter cells. While overnight growth in YNB did produce some hyphal forms, they were not predominant and contributed little to the overall counts performed by hemacytometer. For growth-rate determination, overnight starter cultures were diluted ten folds into fresh medium and grown for approximately 2 hours to ensure the culture we note in stationary phase of growth.

Evaluation of germination in *Candida* was performed by making a heavy suspension of the test organism in serum or YNB, with or without tetracycline and incubating at 37°C for 30 minutes and 3 hours. Cells are examined at each time point and presence or absence of germ tubes determined by microscopic examination at 100x and 400x. Video microscopy was available to compare images obtained from different culture conditions.

Induction of the *CDR-1* gene under various growth conditions was analyzed by flow cytometry in the green fluorescence channel (FL1) as described in our previous studies [8]. Data were normalized in relation to control values, and fluorescence of treated cells was reported as fold-increase-over control (FIOC). Previous work from our laboratory sought to determine if germinated or hyphal cells provided a stronger fluorescent signal than yeast cells. If the instrument is gated on higher forward scatter values, we found that mean channel fluorescence for high forward scatter particles did not contribute disproportionately to the mean channel fluorescence of the population.

Induction of biofilm formation was measured by creating biofilm on the bottoms of flat-well microtiter plates. Briefly, overnight cultures of the selected test strains of *Candida* were grown in unsupplemented growth media or in media supplemented with 17*β*-estradiol or tetracycline at 30°C or 37°C. Starter cultures of selected yeast strains were diluted 10^−4^-fold and allowed to adhere to well bottoms for 1 hour after which all wells were vigorously washed with a plate washer to remove unbound yeast. Media with or without test additives was replaced in the wells, and biofilm was allowed to develop through overnight incubation at 37°C. At the conclusion of biofilm development, the wells again were exhaustively washed and overlaid with 100 *μ*L of 0.5% glucose and 5 *μ*L of Alimar Blue metabolic indicator. Plates were read spectrophometrically at 30 minute intervals for 2 hours, and the area under the time—absorbance_600_ curve (AUC) was taken as an indication of the amount of biomass adherent to the wells.

Hemolysin production was measured by exposing supernatants of spent *Candida* cultures to a 1% human erythrocyte suspension in phosphate buffered saline, removing unlysed erythrocytes by centrifugation and reading the hemoglobin from lysed erythrocytes in the supernatant fluid by spectrophotometry at 540 nm.

## 3. RESULTS

### 3.1. Effects on fungal growth

**Figure 1:**
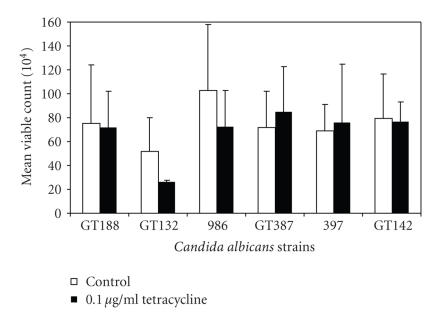
The effect of adding a very low concentration of tetracycline to chemically defined growth medium (YNB) on overnight viable counts of 6 strains of yeast is shown. Three strains of yeast (GT188, 986, and GT132) were previously known to be nonsensitive to 17*β*-estradiol (did not show increased growth yield in the presence of 1 × 10^−5^ M/L 17*β*-estradiol), and 3 strains (GT387, 397, and GT142) were previously shown to be sensitive to growth yield stimulation in the presence of 17*β*-estradiol. Standard deviations from triplicate determinations are shown by error bars. White bars represent growth in YNB, and black bars represent growth in YNB with 0.1 *μ*g/mL tetracycline.

The first question addressed by this study was whether tetracycline could increase fungal growth, since direct growth enhancement could explain why prior studies reported increased yeast colonization after tetracycline therapy. To establish whether any concentration of tetracycline altered growth of *Candida albicans* strains, various concentrations were tested for their effects on overnight growth; concentrations of tetracycline between 5 and 0.15 mg/mL were inhibitory toward *Candida albicans* with 5 mg/mL reducing hemacytometer counts to near zero and 150 *μ*g/mL reducing counts by half compared to the control containing no tetracycline. While hemacytometer counts were useful in distinguishing the obvious difference between tetracycline-treated and control cultures at high tetracycline level, we turned to viable plate counts to elucidate more subtle differences in growth of yeast exposed to sublethal concentrations of the drug. In at least three separate trials with duplicate plate counts (not hemacytometer) determinations, the effect of 0.1 and 0.3 *μ*g/mL proved variable with tetracycline-containing cultures having from 54–126% of control counts. Those organisms previously shown to be stimulated by 17*β*-estradiol showed an insignificant growth increment in the presence of tetracycline, whereas those not stimulated by estrogen did not show increased growth. The magnitude of this tendency was not sufficient to draw firm conclusions about possible growth stimulation by tetracycline (Figure 1).

Because antibiotics may cause fitness-enhancing generalized stress responses at sublethal concentrations and stress responses may be induced rapidly, we also examined viable plate counts at 6 hours in lieu of overnight growth for strain 142. Tetracycline concentrations from 0.3–5 *μ*g/mL increased counts by as much as four folds (Table 1) indicating an effect on growth may be transient but apparently not sustained long enough to produce significantly more growth after overnight culture.

### 3.2. Yeast germination

**Figure 2:**
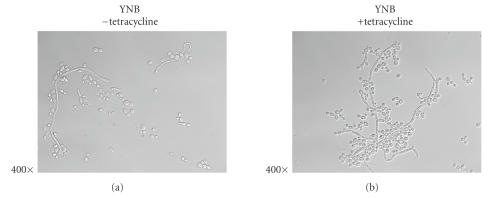
Photomicrographs of *Candida albicans* (Strain GT142) grown overnight at 37°C in YNB or YNB plus tetracycline (5 *μ*g/mL). The yeast tended toward germination and filamentation in YNB, and tetracycline did not inhibit this tendency. Images are representative of many fields that indicate filamentous growth in the presence of tetracycline that is augmented and fungal masses appear larger than in control (YNB) cultures.

**Figure 3:**
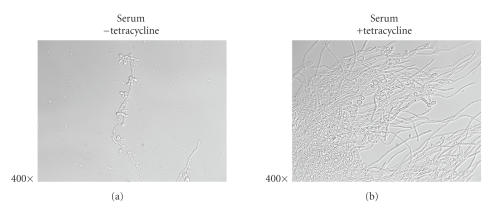
Photomicrographs of *Candida albicans* (Strain GT142) grown overnight at 37°C in serum or serum plus tetracycline (5 *μ*g/mL). The tendency of tetracycline to enhance filamentous growth is particularly evident in the right-hand panel.

The ability of *Candida albicans* to form germ tubes, pseudohyphae, or hyphae is considered an expression of the more virulent forms of the organism. We tested several strains of yeast initially with 0.3 *μ*g/mL of tetracycline in liquid media and human serum to determine if tetracycline altered hyphal production. YNB, a chemically-defined medium, showed the presence of many germ tubes after 3 hours incubation at 37°C, and in human serum, even more extensive germ-tube formation was seen. We were unable to discern an effect of tetracycline on germination in either YNB or serum. These cultures were held at 37°C overnight and viewed again. In YNB, there were mostly yeast forms and a substantial number of pseudohyphae, and a similar morphologic appearance was found among tetracycline-treated YNB cultures. In contrast, culture in serum caused *Candida* transformation to mostly hyphal growth. It was clear that tetracycline did not interfere with hyphal formation at 0.3 *μ*g/mL and may have enhanced hyphal formation. To investigate this further, we tested additional concentrations of tetracycline (1, 3, and 5 *μ*g/mL) and found that at 5 *μ*g/mL it was possible to obtain evidence that in both YNB (Figure 2) and in serum (Figure 3) tetracycline seemed to enhance hyphal formation. The effect was particularly profound when serum was present, a condition possibly more representative of conditions that prevail in vivo.

### 3.3. Regulation of CDR1

In addition to effects on growth, tetracycline might alter the expression of virulence factors related to colonization and symptoms in antibiotic-treated patients. The drug efflux pump *CDR1* has been shown to be promiscuously upregulated by 17*β*-estradiol and coumarin and may be part of a generalized stress response that enhances fitness of *Candida* [8]. With the availability of the CaSA1 organism [8], we were able to examine the effect of tetracycline on *CDR1* expression. The effect on *CDR1* expression was tested by incubation of CaSA 1 with or without tetracycline at 0.1, 0.3, 5, and 10 *μ*g/mL after overnight incubation at 37°C. Flow cytometry was used to determine the relative amount of GFP produced and the known inducer of *CDR1* ; 17*β*-estradiol (at 1 × 10^−5^ M) was used as a positive control. The maximum increase caused by tetracycline was only a 30% increase relative to negative controls (Table 2). This level of induction was significant (*p* = 0.05, unpaired 2-tailed *t*-test) when compared to the unstimulated CaSA 1 organism but was unremarkable compared to the known inducer, estradiol (*p* = 0.0004, 2-tailed unpaired *t*-test versus CaSA 1), especially in view of the fact that the highest molar concentration tetracycline was greater than that of estradiol.

### 3.4. Biofilm

**Figure 4:**
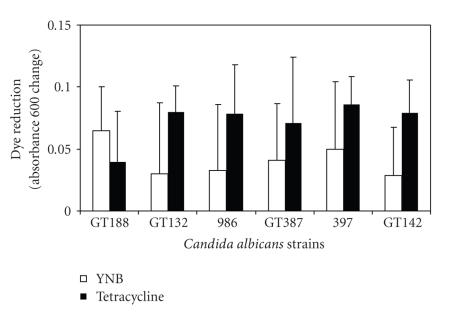
Biofilm was produced in the YNB growth media with or without the addition of tetracycline (0.1 *μ*g/mL). Biofilm was measured by allowing the metabolic indicator Alimar Blue to be reduced by the biomass remaining after vigorous washing of the wells. The relative biofilm measure represents the dye reduction over a 2-hour period measured at 600 nm spectrophotometrically. Of note, 5 of the 6 strains tested showed a tendency toward increased biofilm metabolic activity when exposed to tetracycline, though the variability of the biological assay limited definite conclusions regarding excess biofilm production.

Biofilm is increasingly being recognized as an important contributor to pathogenesis by mucosal organisms. The potential for tetracycline to alter the biofilm production under in vitro culture conditions was examined to gain support for a role of tetracycline in enhancing symptomatic vulvovaginal candidiasis. As illustrated in Figure 4, overnight culture in the presence of tetracycline was not unequivocally associated with increased production of biofilm at 37°C, although a slight, though not statistically different, increase in biofilm metabolic activity was noted for 5 of 6 *Candida* strains tested. It was clear that this biological assay was characterized by substantial variability as seen by the wide standard deviations and perhaps with a refined method, the tendency toward biofilm formation in the presence of tetracycline could be confirmed. In parallel with this study, the effect of nanomolar estradiol induced a nonsignificant trend toward increased level of biofilm activity (data not shown) at 37°C and as such provided data that appeared very similar to tetracycline exposure.

### 3.5. Hemolysin

**Figure 5:**
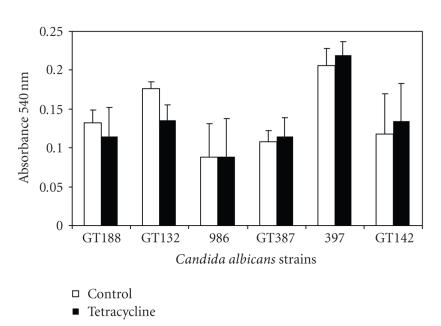
Very little hemolytic activity was detected in cultures of *Candida albicans* regardless of the presence or absence of added tetracycline (0.1 *μ*g/mL). The greatest amount *f* hemolysis detected was 4% lysis of a 1% erythrocyte suspension. As shown by the figure, there was minimal difference between strains employed in this evaluation. It may be concluded that tetracycline has a negligible effect on hemolytic activity of *Candida*.

The final potential virulence property evaluated in this study was *Candida albicans* was hemolysin which may aid the organism in acquiring iron in vivo. Hemolytic activity in spent cultures of *Candida* is typically consistently detected, but the magnitude of effect is relatively low amounting to less than 5% hemolysis of a 1% erythrocyte suspension in 30 minutes. Hemolytic activity is summarized in Figure 5 and shows that baseline hemolytic activity (in the absence of tetracycline or estradiol) varied with strain, and minimal effect was observed when tetracycline (0.1 *μ*g/mL), or nanomolar estradiol was added to the growth medium. Higher concentrations of tetracycline did not appear to affect the hemolytic activity of spent cultures.

## 4. DISCUSSION

This study focused on the possibility that growth of *Candida albicans* and the expression of virulence attributes may be altered by the exposure of this fungal pathogen to tetracycline. A long-standing medical dogma states that antibiotic therapy is often accompanied by development of symptomatic yeast vaginitis, though in a study we conducted during pregnancy we found that antibiotics other than tetracycline did not promote yeast vaginitis. Because an older study of tetracycline [1] linked prolonged treatment with increasing rates of vaginal yeast colonization, we intended to look for direct effects of tetracycline on yeast growth and virulence attribute expression.

Tetracycline, like 17 ß-estradiol, has a phenolic A ring, although the hydroxyl group in tetracycline is in the 2 instead of 3 position as it is in estradiol. Nevertheless, our previous finding that 17 ß-estradiol at concentrations ranging from 1 × 10^−5^ to 1 × 10^−9^ M/L increases *Candida* growth in some strains, and certain virulence attributes are elevated by 17 ß-estradiol, encouraged the current study since it was not clear precisely what chemical signature accounted for some of the previously observed direct effects of 17 ß-estradiol on yeast including growth stimulation and upregulation of drug efflux mechanisms.

Because many biological effects of 17*β*-estradiol have been observed in *Candida albicans* [6–11], we reasoned that tetracycline may have chemical similarities that could elicit similar direct effects on the organism such as increasing growth or upregulating virulence factors. These biological effects could help explain the role of tetracycline in symptomatic yeast vaginitis in a manner that goes beyond the medical dogma that attributes vaginal infection to bacterial killing and simultaneous fungal sparing. However, tetracycline does appear to have antifungal activity, at least when present in high concentration as noted in the present study, raising new questions about old beliefs about pathogenesis of vaginal candidiasis.

After submitting the present manuscript we became aware that White’s group published an important study [12] that indicated that tetracycline was inhibitory toward *Candida, Cryptococcus*, and *Aspergillus*. However, their research focused on in vitro tetracycline concentrations that were 1-2 orders of magnitude larger than the levels investigated in this paper. Still, it is important to realize that tetracycline, while not used as an antifungal drug, does have inhibitory properties that are dose related.

In the current study, we focused on tetracycline concentrations in the range of 0.1–10 *μ*g/mL (2.1 × 10^−7^ M to 2.1 × 10^−5^ M) which is in the range of typical serum concentrations among individuals being treated [13]. At higher concentrations (3–5 mg/mL), tetracycline proved to be antibiotic toward *Candida albicans*, particularly when the yeast strains were cultivated overnight with the drug. A slight increase in growth was obtained with incubation times shorter than overnight with submicrogram/mL concentrations among some, but not all, yeast strains. Moreover, the magnitude of growth increases was quite small and not sufficient to support the hypothesis that growth stimulation by tetracycline was the likely mechanism predisposing to yeast vaginitis. We noted that the tendency of growth effects of tetracycline seemed to mirror those we had previously seen for estradiol, underscoring our belief that as a chemical entity, tetracycline might have some biological effects similar to estradiol. These growth studies may deserve additional evaluation in the future.

Although preliminary growth studies suggested that *Candida albicans*, like bacteria, may be adversely affected by high levels of tetracycline, and the small magnitude of growth stimulation at low concentrations makes direct growth stimulation an unlikely sole explanation for increased vaginitis among tetracycline treated women. However, a growth increment coupled with elevated virulence of yeast offers a potential explanation for tetracycline promotion of symptomatic yeast infection.

Four-virulence attributes were studied mainly at the phenotypic level and appeared to be affected variously by exposure of the yeast to tetracycline. Most striking among these was the finding that germination and related transition from yeast to hyphal growth were augmented in the presence of tetracycline, and this was especially true when serum was present. This finding has practical relevance to the potential effects of tetracycline in vivo because the hyphal form of the organism is reputed to be its most virulent state. Had this study indicated that tetracycline prevents hyphal transformation instead of augmenting it, it would be difficult to suggest how tetracycline treatment might contribute to yeast vaginitis.

For sometime, we have hypothesized that the drug efflux pump *CDR1* may serve the organism to enhance its fitness in hostile environments [11]. To examine the effect of tetracycline on CDR1 expression, a special recombinant indicator strain was employed which explains why this experiment did not involve the panel of test organisms used in the remainder of the study. The yeast CaSA1 produces green fluorescent protein in response to azole drugs and exposure to estradiol [8]; and when sublethal concentrations of tetracycline were introduced to cultures of CaSA1, it was found that a slight (30%) increase in fluorescence occurred at 37°C at 10 *μ*g/mL, but not at lower levels. In the recent study from White’s group [12], they failed to find upregulation of *CDR1* at the levels of tetracycline they employed. Higher levels of tetracycline are likely to suppress protein synthesis as suggested by our growth studies *CDR1* expression that was not studied at concentrations of tetracycline that inhibited fungal growth. Because *CDR1* expression appears to be adaptive under adverse conditions, this factor could incrementally help the yeast to survive under hostile conditions; although as demonstrated by our positive controls, the upregulation of *CDR1* in the presence of tetracycline was far below levels attained with equimolar levels of estradiol (5 *μ*g/mL tetracycline was approximately equal to the molarity of the estradiol positive control).

The virulence attributes of biofilm and hemolysin production were also examined, and there was a tendency toward increased biofilm formation which, though not significant in magnitude, was at least fairly consistent across test strains evaluated. There was clearly a notable degree of variability (see Figure 4) among replicate tests for biofilm production, but because it was a biological rather than strictly analytical method, the variability was not surprising, but did undermine our ability to unequivocally demonstrate tetracycline as a stimulant of biofilm.

Hemolysin, as shown by Figure 5 which in *Candida*, is poorly characterized, tended to be slightly increased in the presence of tetracycline, but the effect was modest and consequently unlikely to have biological significance. It is difficult to claim with certainty from this study that any tendency of women to develop vaginitis symptoms after tetracycline therapy is due to direct effects of the drug on biofilm or hemolysin, but the slight effects on early growth and more profound effects on hyphal formation could contribute to yeast vaginitis.

It is worth noting that a plethora of genes in *Candida* have been identified as having an association with virulence. This list is large and continues to grow, but importantly available molecular methods for analysis of expression of virulence genes could be applied to *Candida* exposed to tetracycline. We have not yet delved into studies of expression of these specific genes because we preferred in this preliminary work, to establish a sufficient biological cause on a phenotypic level to warrant additional studies of putative virulence genes.

We conclude from this investigation that tetracycline, though displaying an antifungal effect at high concentrations, has a small be detectable stimulating effect on growth rate at sublethal concentrations. This effect in conjunction with the more potent is the effect of tetracycline on hyphal morphogenesis, and possible enhancement of biofilm formation may contribute to the tetracycline's relationship to symptomatic yeast vaginitis. While not unequivocally demonstrating that tetracycline affects *Candida* and thereby predisposing to vaginitis, it does suggest that future research should pursue this hypothesis.

Finally, we return to the medical dogma that prompted this report. The concept that vaginal symptoms developing after tetracycline therapy is merely the result of reduction in bacterial populations with a simple overgrowth of yeast is commonly repeated. This explanation, in light of the current study, appears too simplistic as high levels of tetracycline inhibit *Candida* which should reduce the risk of vaginitis, but antifungal concentrations may not be attainable with typical doses. In contrast, sublethal concentrations appear to affect the physiology of the yeast in a way consistent with predisposition to symptomatic yeast vaginitis which may modify the view of pathogenesis of vaginitis following tetracycline treatment.

## Figures and Tables

**Table 1:** Effect of tetracycline in YNB on growth of strain 142 at 6 hours (37°C).

Tetracycline concentration (*μ*g/mL)	Viable plate count (average of duplicate platings)
0	4.2 × 10^4^
0.3	4.7 × 10^4^
0.6	8.4 × 10^4^
1.3	7.6 × 10^4^
2.5	7.1 × 10^4^
5	1.8 × 10^5^
10	6.0 × 10^4^

**Table 2:** Flow cytometry evaluation of CDR1 expression in response tetracycline exposure.

Condition	Fl-1hour (mean channel fluorescence)	FIOC (fold increase over control fluorescence)
CaSA1 (organism background)	5.07 ± 0.88	1.0
Estradiol (positive control)	34.56 ± 4.27	6.86 ± 0.39
10 *μ*g/mL tetracycline	6.52 ± 0.26	1.31 ± 0.23
5 *μ*g/mL tetracycline	5.54 ± 0.79	1.10 ± 0.13
0.3 *μ*g/mL tetracycline	4.12 ± 0.25	0.84 ± 0.19
0.1 *μ*g/mL tetracycline	3.77 ± 0.11	0.76 ± 0.13
